# Effect of Glucagon-Like Peptide-1 (GLP-1) Receptor Agonists and Sodium-Glucose Cotransporter-2 (SGLT-2) Inhibitors on Atherosclerotic Cardiovascular Risk Among Patients With Type 2 Diabetes in Primary Health Care Settings in Jeddah, Saudi Arabia

**DOI:** 10.7759/cureus.103507

**Published:** 2026-02-12

**Authors:** Rahaf A‎ljohani, Riyam Aldubi, Inas Bagabas, Dalia Al-Ghamdi, Ohoud Balkhair, Lujainah Basubrain, Lama Rammal

**Affiliations:** 1 Family Medicine, Ministry of the National Guard–Health Affairs, Riyadh, SAU; 2 Family Medicine, King Abdulaziz Medical City, Jeddah, SAU; 3 Family Medicine, King Fahad Medical City, Riyadh, SAU; 4 Family Medicine, Ministry of the National Guard–Health Affairs, Jeddah, SAU; 5 Family Medicine, King Abdullah International Medical Research Center, Jeddah, SAU; 6 Medicine, King Saud bin Abdulaziz University for Health Sciences, Jeddah, SAU

**Keywords:** ascvd risk, atherosclerotic cardiovascular disease, cardiovascular outcomes, dapagliflozin, glp-1 receptor agonists, primary health care, saudi arabia, semaglutide, sglt-2 inhibitors, type 2 diabetes mellitus

## Abstract

Background

Saudi Arabia has one of the highest global prevalences of diabetes mellitus (39.5%), with cardiovascular disease (CVD) representing the leading cause of mortality among patients with diabetes. Sodium-glucose cotransporter 2 (SGLT-2) inhibitors (e.g., dapagliflozin) and glucagon-like peptide-1 receptor agonists (GLP-1RAs; e.g., semaglutide) have demonstrated cardiorenal benefits; however, their effect on 10-year atherosclerotic cardiovascular disease (ASCVD) risk in Saudi populations remains underexplored.

Methods

This retrospective cohort study included 196 adults aged 40 to 79 years with type 2 diabetes who were prescribed dapagliflozin or semaglutide at King Saud Bin Abdulaziz Medical City between March 2021 and March 2022. Patients with type 1 diabetes, established CVD, or chronic kidney disease (estimated glomerular filtration rate <60 mL/min/1.73 m²) were excluded. Data on demographic characteristics, metabolic parameters (HbA1c, lipid profile, estimated glomerular filtration rate), and cardiovascular events were collected at baseline and at six, nine, and 12 months. Statistical analyses were performed using paired t-tests and McNemar’s test and IBM SPSS Statistics for Windows, Version 28 (Released 2021; IBM Corp., Armonk, New York, United States).

Results

The mean age of the studied population was 57.4±11.3 years, and 70.9% were female participants. Significant improvements were observed in HbA1c levels (from 8.9±1.1% to 7.4±0.8%; P<0.001) and systolic blood pressure (from 137±17 to 131±17 mmHg; P=0.004). Estimated glomerular filtration rate showed a modest increase (from 91±18 to 92±18 mL/min/1.73 m²; P<0.001), while albuminuria remained unchanged. Rates of cardiovascular events, including myocardial infarction and stroke, did not differ significantly during follow-up (P>0.05).

Conclusions

Treatment with dapagliflozin and semaglutide was associated with significant improvements in glycemic control and systolic blood pressure but was not associated with a reduction in short-term cardiovascular events in this high-risk cohort. Long-term studies are warranted to assess the impact of these agents on ASCVD risk reduction in this population.

## Introduction

Diabetes mellitus (DM) is a chronic disease characterized by elevated blood glucose levels and is one of the most prevalent conditions worldwide [1]. According to the International Diabetes Federation, Saudi Arabia ranked 16th globally for diabetes prevalence in 2022. Epidemiological studies report that the prevalence of DM in Saudi Arabia has increased dramatically from 8.5% to 39.5% over the past 30 years [2]. This growing burden is associated with high morbidity and mortality [3]. Diabetes contributes to complications affecting the cardiovascular, renal, nervous, and visual systems, with atherosclerotic cardiovascular disease (ASCVD) being the leading cause of death among affected patients.

Traditional cardiovascular risk factors, including hypertension, cigarette smoking, dyslipidemia, and obesity, are highly prevalent in patients with diabetes and further amplify cardiovascular risk [4]. Evidence from the Multiple Risk Factors Intervention Trial (MRFIT) confirmed that the presence of even one of these risk factors markedly increases cardiovascular mortality in men with diabetes compared with those without diabetes [4]. Similarly, a cross-sectional study from Al Khobar, Saudi Arabia, found that 89% of patients with diabetes had two or more cardiovascular risk factors in addition to diabetes [5]. A systematic review estimated that approximately one-third of patients with type 2 diabetes mellitus (T2DM) worldwide develop cardiovascular disease, with coronary artery disease (CAD) and stroke being the most common manifestations [6].

Therapeutic advances such as sodium-glucose cotransporter 2 (SGLT-2) inhibitors and glucagon-like peptide-1 receptor agonists (GLP-1RAs) have shown promising results in reducing diabetes-related complications [3]. Both drug classes have demonstrated cardiovascular and renal protection in major clinical outcome trials, with reductions in hospitalization for heart failure and all-cause mortality [3].

Dapagliflozin, an SGLT-2 inhibitor, has consistently been associated with reductions in body weight and systolic blood pressure, while maintaining a favorable safety profile [7]. In addition, it may reduce the risk of microvascular complications such as diabetic kidney disease and retinopathy [8]. The Dapagliflozin Effect on Cardiovascular Events-Thrombolysis in Myocardial Infarction 58 (DECLARE-TIMI 58) trial demonstrated a 17% reduction in the composite outcome of cardiovascular death or hospitalization for heart failure with dapagliflozin [9].

Semaglutide, a GLP-1RA, has shown renoprotective benefits, particularly through reducing albuminuria in patients with microalbuminuria and macroalbuminuria compared with placebo [3]. However, its effects in patients with chronic kidney disease (CKD) remain less well studied. Current evidence suggests that patients with high cardiovascular risk may particularly benefit from GLP-1RAs, whereas those with CKD or heart failure may derive greater benefit from SGLT-2 inhibitors [10].

In a landmark randomized clinical trial, published in 2019, dapagliflozin reduced the rate of cardiovascular death or hospitalization for heart failure compared with placebo (4.9% vs. 5.8%) [9]. Furthermore, GLP-1RAs, including semaglutide, have demonstrated significant reductions in major adverse cardiovascular events (MACE) in patients with T2DM, including those without established cardiovascular disease [11,12].

This study, conducted at King Abdulaziz Medical City, Jeddah, Saudi Arabia, aims to evaluate changes in 10-year ASCVD risk among patients with T2DM treated with dapagliflozin or semaglutide over a one-year follow-up period. To our knowledge, this is the first study in Jeddah to assess both agents in routine clinical practice, providing valuable insight into their potential for cardiovascular risk reduction in Saudi patients.

## Materials and methods

Study design and setting

This retrospective cohort study was conducted at the specialized poly-clinic for primary health care center (SPC-PHC) at the King Saud Bin Abdulaziz Medical City in Jeddah, Saudi Arabia. Patient records from March 2021 to 2022 were included.

Study population

The study population comprised adult patients aged 40 to 79 years with a diagnosis of T2DM who were prescribed either semaglutide or dapagliflozin during the observation period. Exclusion criteria were type 1 diabetes mellitus; established cardiovascular disease (including myocardial infarction, stroke, or severe heart failure); chronic kidney disease (defined as an estimated glomerular filtration rate (eGFR)<60 mL/min/1.73 m² or receipt of dialysis); current systemic corticosteroid therapy; psychiatric conditions potentially impairing treatment adherence; pediatric patients; and pregnancy.

Sample size

Sample size estimation was performed using Raosoft® software (Raosoft Inc., Seattle, WA, US) with a 95% confidence level, 50% estimated prevalence, and a 5% margin of error. The calculated minimum sample size was 313 patients from a total pool of 524 eligible cases. However, all qualifying patients from the specified timeframe were included to maximize statistical power and ensure data completeness.

Data collection

Clinical data were extracted by trained research staff through a comprehensive chart review using the electronic health record system. Variables collected included demographic characteristics (age, sex, race), clinical parameters (blood pressure, lipid profile, smoking status), laboratory values (HbA1c, eGFR, albumin-to-creatinine ratio), medication regimens, and documented cardiovascular outcomes. Longitudinal follow-up data at six, nine, and 12 months after treatment initiation were obtained for outcome assessment.

Statistical analysis

Continuous variables are summarized as means ± standard deviations (SDs) or medians with interquartile ranges (IQR), as appropriate. Categorical variables are presented as frequencies and percentages. Normality of continuous variables was assessed using the Shapiro-Wilk test and visual inspection of Q-Q plots.

Paired comparisons were performed between baseline and 12-month values to evaluate treatment effects over time. For normally distributed continuous variables, paired t-tests were used; for non-normally distributed variables, the Wilcoxon signed-rank test was applied. Changes in categorical outcomes, including cardiovascular events, were assessed using the McNemar’s test for paired nominal data.

All statistical tests were two-tailed, with a P value <0.05 considered statistically significant. Analyses were conducted using IBM SPSS Statistics for Windows, Version 28 (Released 2021; IBM Corp., Armonk, New York, United States).

## Results

Baseline characteristics

A total of 196 patients with T2DM receiving either a GLP-1RA or an SGLT-2 inhibitor were included in the analysis. The mean (±SD) age of the cohort was 57.4 ± 11.3 years, and the majority were women (139 patients, 70.9%). The mean body mass index (BMI) was 33.0 ± 6.6 kg/m², indicating a predominantly obese population. The mean duration of diabetes was 12.8 ± 7.4 years (data available for 170 patients).

Hypertension was present in 132 patients (67.3%) and dyslipidemia in 178 patients (90.8%). Only 13 patients (6.6%) were current smokers. The use of cardiovascular preventive therapies was common, with 45 patients (23.0%) taking aspirin and 172 patients (87.8%) receiving a statin (Table 1).

**Table 1:** Baseline characteristics of the study population Baseline characteristics of patients with type 2 diabetes mellitus receiving either a glucagon-like peptide-1 (GLP-1) receptor agonist or an sodium glucose cotransporter-2 (SGLT-2) inhibitor. Continuous variables are presented as mean ± standard deviation, and categorical variables are presented as number (percentage). ^†^Duration of diabetes was available for 170 patients.

Characteristic	Overall (N = 196)
Age (yrs; mean ± SD)	57.4 ± 11.3
Female sex (n; %)	139 (70.9)
Body Mass Index (kg/m²; mean ± SD)	33.0 ± 6.6
Duration of diabetes (yrs; mean ± SD)^†^	12.8 ± 7.4
Hypertension (n; %)	132 (67.3)
Dyslipidemia (n; %)	178 (90.8)
Current smoker (n; %)	13 (6.6)
Uses aspirin (n; %)	45 (23.0)
Uses statin (n; %)	172 (87.8)

Use of antidiabetic medications

In addition to treatment with a GLP-1RA or an SGLT-2 inhibitor, most patients were prescribed other glucose-lowering medications. Metformin was used by 168 patients (85.7%), and 81 patients (41.3%) were receiving gliclazide. A smaller proportion were taking sitagliptin (35.2%), basal insulin (glargine) (58.2%), or multiple daily insulin injections (46.9%). Use of pioglitazone (5.1%) and acarbose (0.5%) was infrequent. Many patients were on more than one antidiabetic agent concurrently (Table 2).

**Table 2:** Baseline use of antidiabetic medications other than GLP-1 receptor agonists or SGLT-2 inhibitors Medication use reported at baseline. All patients were receiving either a glucagon-like peptide-1 (GLP-1) receptor agonist or an sodium-glucose transport 2 (SGLT-2) inhibitor as part of the inclusion criteria. Patients may have been on more than one medication concurrently. Percentages are based on a total of 196 patients.

Medication	No. of patients (%)
Metformin	168 (85.7)
Gliclazide	81 (41.3)
Acarbose	1 (0.5)
Pioglitazone	10 (5.1)
Sitagliptin	69 (35.2)
Glargine (basal insulin)	114 (58.2)
Multiple daily insulin doses	92 (46.9)

Changes in clinical parameters over 12 months

Significant improvements were observed in glycemic control and renal function over the 12-month follow-up period. The mean HbA1c decreased from 8.9 ± 1.1% at baseline to 7.4 ± 0.8% at 12 months (P<0.001). Systolic blood pressure declined from 137 ± 17 mmHg to 131 ± 17 mmHg (P=0.004), while diastolic blood pressure remained unchanged (76 ± 10 mmHg at baseline vs. 75 ± 9 mmHg at 12 months, P=0.355).

There were no significant changes in lipid parameters, including low-density lipoprotein (LDL), high-density lipoprotein (HDL), and triglycerides, over the study period. The eGFR improved modestly from 91 ± 18 to 92 ± 18 mL/min/1.73 m² (P<0.001). The albumin-creatinine ratio (ACR) fluctuated over time but did not show a statistically significant change from baseline to 12 months (P=0.633) (Table 3).

**Table 3:** Change in clinical parameters over 12 months P values represent results of paired t-tests comparing baseline values with those at 12 months. Values are presented as mean ± standard deviation. SBP, systolic blood pressure; DBP, diastolic blood pressure; eGFR, estimated glomerular filtration rate.

Parameter	Baseline	6 months	9 months	12 months	P value
Glycated hemoglobin (HbA1c), %	8.9 ± 1.1	7.8 ± 0.9	7.6 ± 1.0	7.4 ± 0.8	<0.001
Systolic blood pressure, mmHg	137 ± 17	131 ± 15	135 ± 15	131 ± 17	0.004
Diastolic blood pressure, mmHg	76 ± 10	76 ± 9	76 ± 13	75 ± 9	0.355
Low-density lipoprotein (LDL), mmol/L	2.6 ± 0.9	2.5 ± 0.9	2.5 ± 0.7	2.6 ± 0.9	0.979
High-density lipoprotein (HDL), mmol/L	1.1 ± 0.3	1.2 ± 0.9	1.1 ± 0.3	1.2 ± 0.3	0.972
Triglycerides, mmol/L	1.7 ± 1.0	1.6 ± 1.1	1.7 ± 1.2	1.5 ± 0.7	0.254
eGFR, mL/min/1.73 m²	91 ± 18	89 ± 19	89 ± 18	92 ± 18	<0.001
Albumin–creatinine ratio, mg/g	5.1 ± 8.6	4.6 ± 7.6	1.8 ± 1.6	6.4 ± 14.9	0.633

Cardiovascular events during follow-up

Cardiovascular outcomes remained relatively stable over the 12 months. The number of patients with prior myocardial infarction remained unchanged (14 (7.1%) at both baseline and 12 months), as did the rates of stroke (seven (3.6%) vs. eight (4.1%), P=1.000), peripheral vascular disease (2.0% vs. 2.6%, P=1.000), and heart failure (1.0% vs. 2.0%, P=0.500). None of these differences reached statistical significance (Table 4).

**Table 4:** Prevalence of cardiovascular conditions at baseline and at 12 months P values were calculated using McNemar’s test for paired nominal data. Values represent the number and percentage of patients (n=196) with each condition at baseline and at the 12-month follow-up. Percentages may not sum to 100 due to rounding.

Condition	Baseline (n; %)	12 Months (n; %)	P Value
Myocardial infarction	14 (7.1%)	14 (7.1%)	1.000
Stroke	7 (3.6%)	8 (4.1%)	1.000
Peripheral vascular disease	4 (2.0%)	5 (2.6%)	1.000
Heart failure	2 (1.0%)	4 (2.0%)	0.500

## Discussion

In this retrospective cohort study of adults with T2DM treated with either a GLP-1RA or an SGLT-2 inhibitor, we observed significant improvements in glycemic control and modest reductions in systolic blood pressure over 12 months. The mean HbA1c decreased substantially, highlighting the effectiveness of these therapies in real-world clinical practice. Renal function, as measured by eGFR, showed slight improvement, suggesting potential renal benefits or at least stabilization over the follow-up period. Lipid profiles remained largely unchanged, and diastolic blood pressure did not show significant variation. Cardiovascular outcomes - including myocardial infarction, stroke, peripheral vascular disease, and heart failure - remained stable over the study period.

The observed improvements in glycemic control are consistent with the mechanisms of action of GLP-1RAs, which enhance glucose-dependent insulin secretion and delay gastric emptying, and SGLT-2 inhibitors, which promote urinary glucose excretion [10,11]. The modest reduction in systolic blood pressure may reflect the natriuretic and diuretic effects associated with SGLT-2 inhibitors, as well as weight reduction and improved metabolic profiles with GLP-1RAs [10]. Even small reductions in blood pressure can be clinically meaningful for long-term cardiovascular risk.

Renal outcomes are particularly relevant given the high prevalence of diabetic kidney disease. Stabilization or slight improvement in eGFR over 12 months suggests that these therapies may have renal-protective effects, consistent with prior evidence for SGLT-2 inhibitors and GLP-1RAs in slowing kidney function decline [3,9].

Cardiovascular events remained stable over the 12-month period, which is expected given the relatively short duration and the sample size. The development of ASCVD is typically long-term, and meaningful changes in event rates are unlikely to be detected in this timeframe. However, the absence of increased adverse events supports the short-term cardiovascular safety of these therapies [7,8].

Clinical implications

Our findings support the use of GLP-1RAs and SGLT-2 inhibitors as effective options for improving glycemic control and modestly reducing cardiovascular risk factors in real-world clinical practice. Many patients in the cohort were on multiple antidiabetic agents concurrently, reflecting the complex management often required in long-standing T2DM. The combination of therapies targeting different mechanisms of glucose regulation appears effective and tolerable, reinforcing guideline-based approaches for individualized, multifactorial treatment [10,11].

Additionally, the observed improvement in renal function, even if modest, may be clinically meaningful over longer durations, given the high risk of chronic kidney disease progression in this population. These findings underscore the potential role of GLP-1RAs and SGLT-2 inhibitors in comprehensive cardiometabolic management beyond glycemic control alone [3,9].

Strengths and limitations

This study has several notable strengths. The use of real-world clinical data from a major tertiary care center enhances generalizability to similar populations. The longitudinal design with standardized follow-up at six, nine, and 12 months allowed for robust assessment of temporal changes in metabolic and cardiovascular parameters. Comprehensive data collection captured multiple cardiovascular risk factors simultaneously, including glycemic control, renal function, lipid profiles, and documented cardiovascular events. Statistical analyses, including paired comparisons and adjustment for baseline characteristics, strengthened the validity of our findings despite the observational design.

Several limitations should be acknowledged. The final sample size of 196 patients was smaller than the initially calculated target of 312, potentially limiting the ability to detect modest differences in cardiovascular outcomes, although post-hoc analyses confirmed adequate power for primary metabolic endpoints. The 12-month follow-up period may be insufficient to capture long-term cardiovascular benefits or risks. As a retrospective, single-center study, residual confounding from unmeasured factors such as medication adherence, diet, and physical activity cannot be excluded. Finally, the findings may not be generalizable to other healthcare settings or more diverse patient populations.

Future directions

Future research should involve larger, multicenter cohorts with longer follow-up periods to fully evaluate cardiovascular and renal outcomes. Prospective studies incorporating standardized outcome assessments, patient-reported measures, and adherence monitoring would strengthen the evidence base. Additionally, examining the differential effects of GLP-1RAs versus SGLT-2 inhibitors, as well as their combination, could inform optimized treatment strategies for patients with T2DM [11,12].

## Conclusions

This real-world study demonstrates that GLP-1RAs and SGLT-2 inhibitors are effective in improving glycemic control and modestly reducing systolic blood pressure in adults with T2DM. Renal function remained stable, and cardiovascular outcomes did not worsen over the 12-month follow-up, supporting the short-term safety and clinical utility of these therapies. These findings highlight the potential of both drug classes as key components of individualized, multifactorial diabetes management, while emphasizing the need for longer-term studies to fully evaluate their cardiovascular and renal benefits in diverse patient populations.
